# Supplementary material for: DNA repair by Rad52 liquid droplets
Source: Nat Commun. 2020 Feb 4;11:695. doi: 10.1038/s41467-020-14546-z (PMC7000754; doi:10.1038/s41467-020-14546-z)
Supplement: Supplementary file 1 — Supplementary Information [file 41467_2020_14546_MOESM1_ESM.pdf]

Supplementary Information for
DNA repair by Rad52 liquid droplets

Oshidari *et al.*

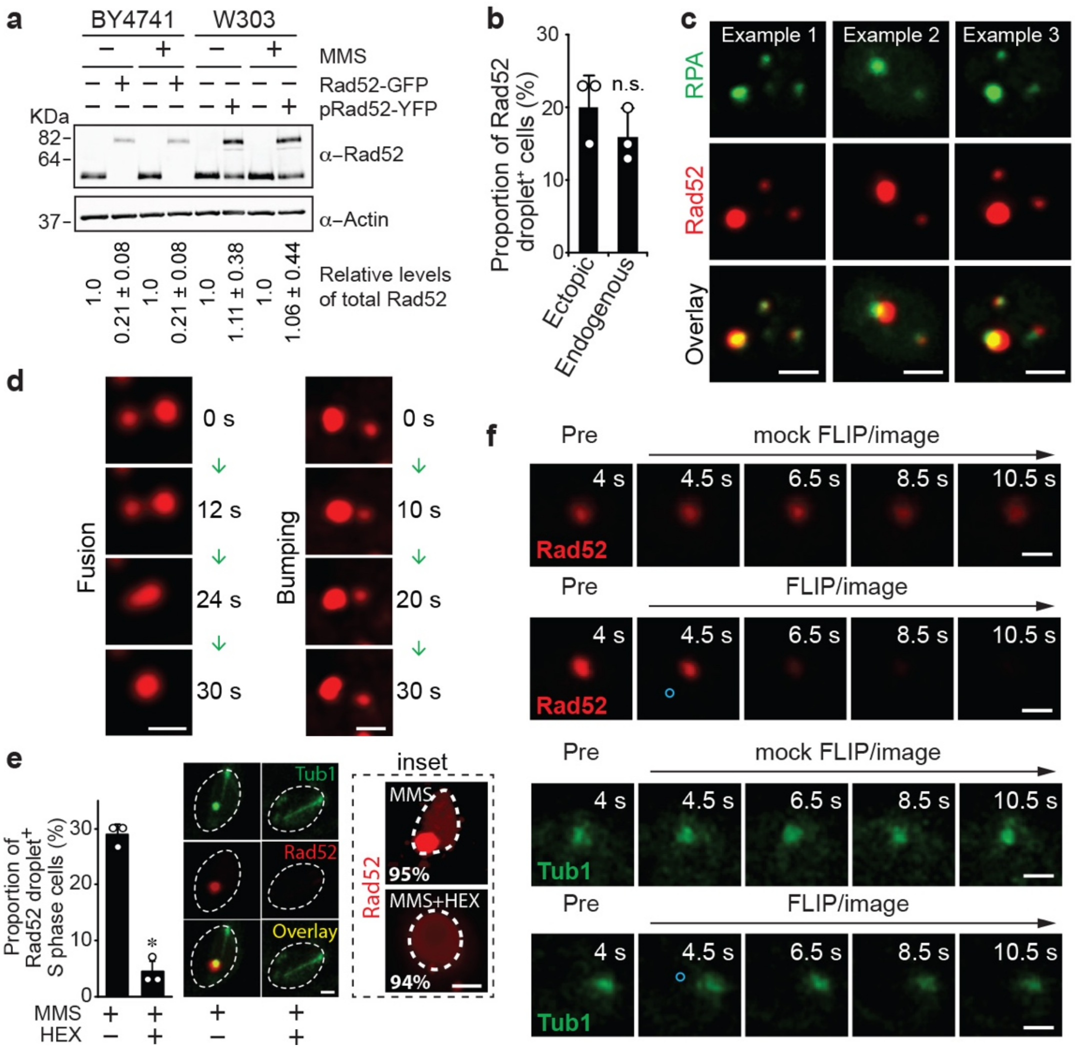

Supplementary Figure 1│ DSB-indicating Rad52 foci behave like liquid droplets *in vivo.* a-b, Plasmid-borne expression of fluorescently labelled Rad52, but not endogenously tagged Rad52, preserves total Rad52 protein levels (**a**) and MMS-induced Rad52 focus formation is similar when Rad52 is endogenously or exogenously tagged (**b**). Quantifications and standard deviations of total Rad52 protein levels in each lane normalized to its respective untagged control from three independent experiments are presented below the blots. **c**, Live cell microscopy shows that all Rad52 foci observed associate with an RPA focus (n=3, 251 foci). **d**, Zeocin-induced Rad52 droplets exhibit liquid droplet behaviours (scale bar, 0.5 μM). **e**, 1,6-Hexanediol disrupts Rad52 liquid droplets (n=3, 120 cells). Inset: 1,6-hexanediol disrupts MMS-induced Rad52 foci without altering the percentage of Rad52 fluorescence that is associated with the nucleus (dashed outline). (scale bar, 0.5 μm). **f**, Representative images of Rad52-YFP and GFP-Tub1 signal loss during FLIP experiments. Scale bars, 1 μM. Quantifications represent the mean ± s.d.; *$P < 0.0001$ in χ^2 test. Source data are provided as a Source Data file.

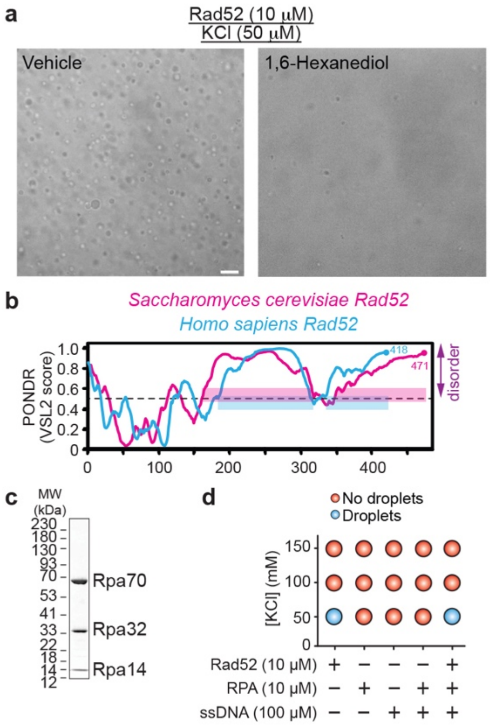

Supplementary Figure 2│ Factors that influence Rad52 phase-separation in *vitro*. a, Addition of 1,6-Hexanediol disrupts pre-formed Rad52 liquid droplets *in vitro*. Scale bar, 10 µm. **b**, Predictor of Natural Disordered Regions (PONDR) reveals that yeast and human Rad52 display similar disorder profiles across their amino acid sequences. **c-d**, Addition of RPA in complex with ssDNA does not influence Rad52 phase separation at varying salt concentrations. Source data are provided as a Source Data file.

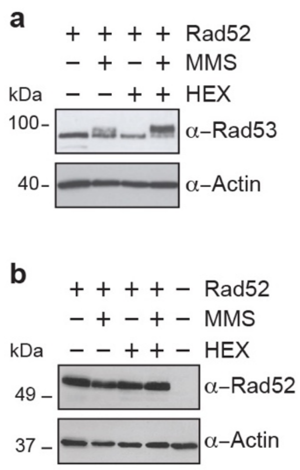

Supplementary Figure 3│ Phase-separation disruptor 1,6-Hexanediol hyper-activates the DNA damage checkpoint only in the presence of DNA damage induction. a-b, Treatment with 1,6-Hexanediol (HEX) leads to hyperactivation of the Rad53 DNA damage checkpoint protein only in the prescence of MMS (**a**) without altering cellular Rad52 protein levels (**b**). Source data are provided as a Source Data file.

a

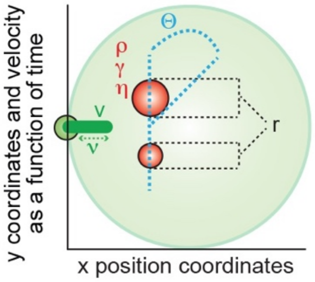

b

Physical Properties	Calculated (C) or Tested (T)	Parameter values
Droplet size ratio (r)	C/T	**0.46**
Droplet density (ρ)	C/T	**1020** μg/μL
Droplet Surface tension (γ)	T	1, 10, 100 μN/m
Droplet viscosity (η)	T	**0.005**, 0.5, 10 Pa s
Angle range to y-axis (Θ)	C/T	0, 90 degrees
Intermitant MT velocity (v)	C/T	20, 40 nm/s
Frequency of velocity (ν)	T	10, 100, 1000 Hz

Supplementary Figure 4│ Parameters of CFD simulations of Rad52 liquid droplet fusions. a-b, Schematic (**a**) and list (**b**) illustrating key parameters incorporated in the CFD simulations. Parameter values underlined are compatible with fusion while those bolded result in the most efficient fusion kinetics.

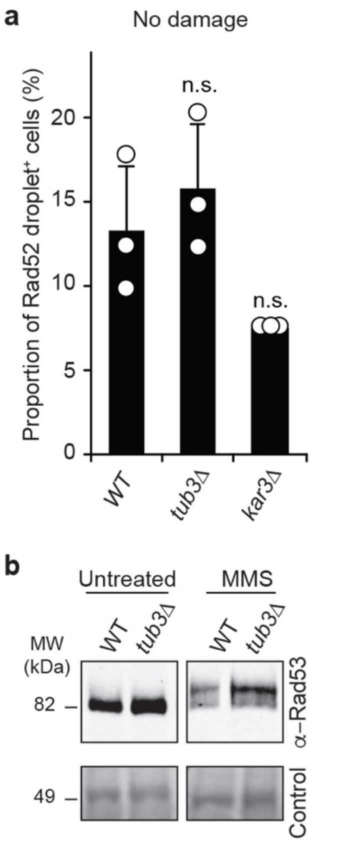

Supplementary Figure 5│ Endogenous levels of Rad52 droplets. a, Quantification of Rad52-YFP droplets via live-cell microscopy. Quantifications represent the mean ± s.d.; χ^2 test was used. **b**, Longer running gel electrophoresis followed by western blotting shows that Tub3 deletion induces a higher migrating band indicating Rad53 hyper-phosphorylation. Ponceau serves as loading control. Source data are provided as a Source Data file.

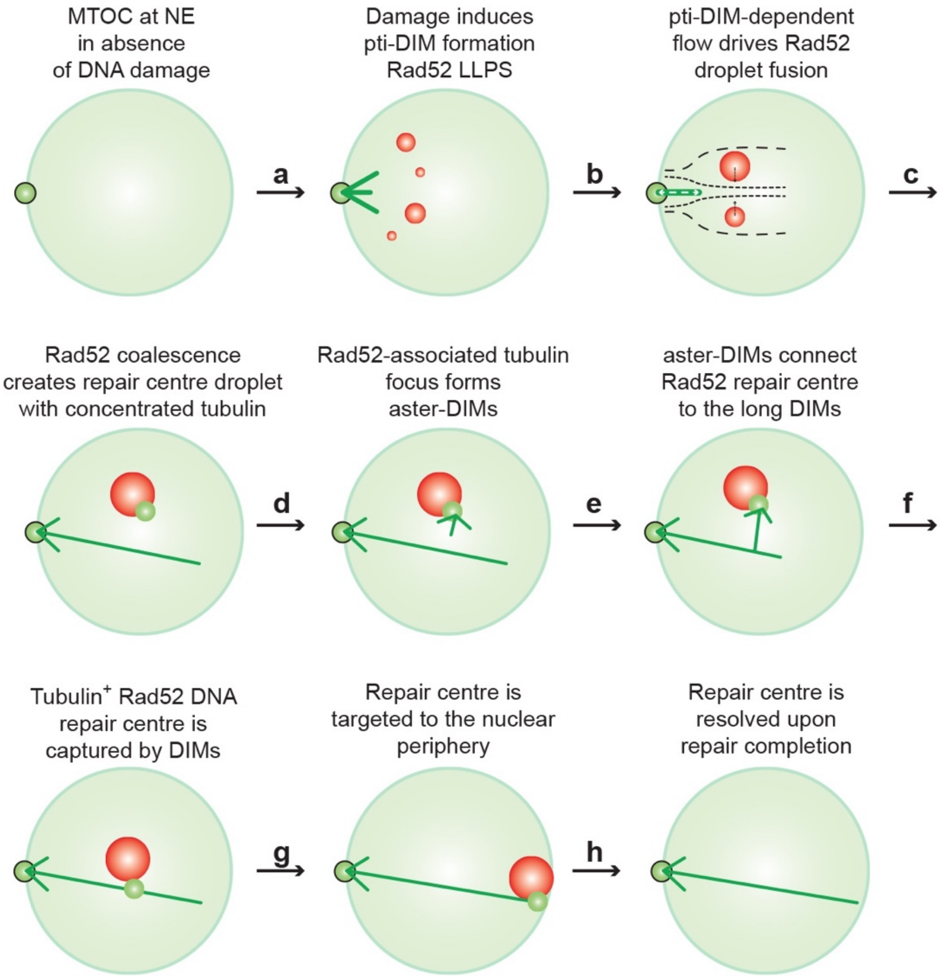

Supplementary Figure 6 | Model. Upon the induction of multiple DNA breaks, the evolutionarily conserved Rad52 protein assembles liquid droplets (red spheres) at the sites of DNA damage across the nucleus (large faint green sphere) (**a**). Novel intranuclear filaments termed pti-DIMs (short green sticks) also emerge from the nuclear envelope-embedded MTOC (**a**). Extension-shortening motions of pti-DIMs can then create a nucleoplasmic flow driving the fusion of the separate Rad52 droplets into a DNA repair centre droplet (**b**, **c**). The repair centre droplet internally concentrates tubulin and projects novel aster-like nuclear filaments termed aster-DIMs (**c**, **d**). One of the latter filaments matures and allows the Rad52 repair centre droplet to be captured by a long DIM (**e**, **f**) for mobilization to the nuclear periphery for repair (**g**, **h**). MTOC, microtubule organizing centre; NE, nuclear envelope; pti-DIMs, petite DNA damage-inducible intranuclear microtubule filaments; LLPS, liquid-liquid phase separation; aster-DIMs, aster-like DNA damage-inducible intranuclear microtubule filaments.

Supplementary Table 1 │ List of S. cerevisiae strains and plasmids used in this study.

Reagent	Genotype	Source
KMY 2559	W303 MATa ade2-1 trp1-1 can1-100 leu2-3,112 his3-11,15 ura3-1 GAL+ psi+ ssd1-d2 RAD5+	This study
KMY 372	BY4741 MATa his3Δ1 leu2Δ0 met15Δ0 ura3Δ0	This study
KMY 3426	W303 MATa ade2-1 trp1-1 can1-100 leu2-3 URA3 GFP-Tub1-HIS3 nup49-GFP-KanMX pKM199-(Rad52-YFP-TRP1)	This study
KMY 3557	BY4741 Rfa1-GFP-HIS3+ pKM201 (Rad52-YFP-LEU2)	This study
KMY 3542	BY4741 Rad52-GFP-HIS3	Kind gift from Brown lab
KMY 3581	KMY 3426 tub3Δ::HphR	This study
KMY 3459	KMY 3426 kar3Δ::HphR	This study
pKM199	Rad52-YFP-TRP1	Kind gift from Durocher lab
pKM 201	Rad52-YFP-LEU2	This study
pKM397	(His)6-RAD52(N+M)::pET11d	Kind gift from Krejci lab
pKM 412	(His)6-RAD52Δ307 (N+M)::pET11d	This study
pKM 403	p11d-sctRPA	Addgene
