# Supplementary material for: DNA repair by Rad52 liquid droplets
Source: Nat Commun. 2020 Feb 4;11:695. doi: 10.1038/s41467-020-14546-z (PMC7000754; doi:10.1038/s41467-020-14546-z)
Supplement: Supplementary file 2 — Description of Additional Supplementary Files [file 41467_2020_14546_MOESM2_ESM.pdf]

Description of Additional Supplementary Files

File Name: Supplementary Movie 1
Description: Live-cell time-lapse confocal microscopy showing multiple Rad52 foci (Rad52-YFP) engaging in dripping behaviour in a representative cell that was treated with 0.03% MMS to induce DNA damage. Scale bar, 1 µm. CL2

File Name: Supplementary Movie 2
Description: Live-cell time-lapse confocal microscopy reveals that Rad52 foci (Rad52- YFP) can fuse with each other in a representative cell that was treated with 0.03% MMS to induce DNA damage. Two fusion events are seen on the right. Scale bar, 1 µm.

File Name: Supplementary Movie 3:
Description: Live-cell time-lapse confocal microscopy showing multiple smaller Rad52 foci (Rad52-YFP) bumping into one larger central droplet in a yeast cell that was treated with 0.03% MMS to induce DNA damage. Scale bar, 1 µm.

File Name: Supplementary Movie 4
Description: Live-cell time-lapse confocal microscopy from a FLIP experiment. Rad52 droplet (Rad52-YFP) intensity rapidly decreases upon photobleaching of a nucleoplasmic point outside the Rad52 focus. Yeast cells were treated with 0.03% MMS to induce DNA damage.

File Name: Supplementary Movie 5
Description: Light microscopy showing the fusion of Rad52 droplets assembled in vitro.

File Name: Supplementary Movie 6
Description: Live-cell time-lapse confocal microscopy revealing dynamic pti-DIMs inside the nucleus. Cells were treated with 0.03% MMS to induce DNA damage. GFP-Tub1 (a-Tubulin) and Nup49-GFP (nuclear pore complexes) are shown in cyan. Scale bar, 1 µm.

File Name: Supplementary Movie 7
Description: Time-lapse from Supplementary Movie 6 overlaid with the corresponding Rad52 droplet signal (Rad52-YFP shown in magenta). Scale bar, 1 µm.

File Name: Supplementary Movie 8
Description: Representative computational flow dynamics simulation in which pti-DIM extension-shortening cycles resulted in the fusion of two Rad52 droplets. The line connecting the droplets in their initial position was perpendicular to the velocity exerted by the pti-DIMs at the centre of the Yaxis. The parameters used in this simulation were r = 046, r = 1014 Kg/m3 , s =1.4 uN/m, µ = 0.005, Q = 90°, u = 41 nm/s, and f = 1000 Hz as shown in Supplementary Figure 2.

File Name: Supplementary Movie 9
Description: Representative computational flow dynamics simulation in which pti-DIM extension-shortening cycles failed to result in the fusion of two Rad52 droplets. The line connecting the droplets in their initial position was rotated clockwise by 20° compared to the position in Supplementary Movie 8, thereby changing Q to 110°. Other simulation parameters were identical to those used in that movie.

File Name: Supplementary Movie 10

Description: Live-cell time-lapse confocal microscopy showing a large Rad52 droplet with an internally concentrated tubulin focus that is captured by and travels along a stable DIM. Cells were treated with 0.03% MMS to induce DNA damage. GFP-Tub1 (tubulin) and Nup49-GFP (nuclear envelope) are shown in cyan and Rad52-YFP in magenta. Scale bar, 1 µm
