# Supplementary material for: DNA repair by Rad52 liquid droplets
Source: Nat Commun. 2020 Feb 4;11:695. doi: 10.1038/s41467-020-14546-z (PMC7000754; doi:10.1038/s41467-020-14546-z)
Supplement: Supplementary file 13 — Reporting Summary [file 41467_2020_14546_MOESM13_ESM.pdf]

nature research

Corresponding author(s): Karim Mekhail, PhD

Last updated by author(s): Jan 9, 2020

Reporting Summary

Nature Research wishes to improve the reproducibility of the work that we publish. This form provides structure for consistency and transparency in reporting. For further information on Nature Research policies, see Authors & Referees and the Editorial Policy Checklist .

Statistics

For all statistical analyses, confirm that the following items are present in the figure legend, table legend, main text, or Methods section.

n/a	Confirmed	
☐	☒	The exact sample size (n) for each experimental group/condition, given as a discrete number and unit of measurement
☐	☒	A statement on whether measurements were taken from distinct samples or whether the same sample was measured repeatedly
☐	☒	The statistical test(s) used AND whether they are one- or two-sided<br>*Only common tests should be described solely by name; describe more complex techniques in the Methods section.*
☐	☒	A description of all covariates tested
☐	☒	A description of any assumptions or corrections, such as tests of normality and adjustment for multiple comparisons
☐	☒	A full description of the statistical parameters including central tendency (e.g. means) or other basic estimates (e.g. regression coefficient) AND variation (e.g. standard deviation) or associated estimates of uncertainty (e.g. confidence intervals)
☐	☒	For null hypothesis testing, the test statistic (e.g. F, t, r) with confidence intervals, effect sizes, degrees of freedom and P value noted<br>*Give P values as exact values whenever suitable.*
☒	☐	For Bayesian analysis, information on the choice of priors and Markov chain Monte Carlo settings
☒	☐	For hierarchical and complex designs, identification of the appropriate level for tests and full reporting of outcomes
☒	☐	Estimates of effect sizes (e.g. Cohen's d, Pearson's r), indicating how they were calculated

Our web collection on statistics for biologists contains articles on many of the points above.

Software and code

Policy information about availability of computer code

Data collection	Simulation data collection was done with ANSYS-Fluent.
Data analysis	Simulation data was processed using Tecplot. Simulation pressures was calculated using MATLAB. Other data were compiled in Excel and statiscally analyzed using Graphpad Prism.

For manuscripts utilizing custom algorithms or software that are central to the research but not yet described in published literature, software must be made available to editors/reviewers. We strongly encourage code deposition in a community repository (e.g. GitHub). See the Nature Research guidelines for submitting code & software for further information.

Data

Policy information about availability of data

All manuscripts must include a data availability statement. This statement should provide the following information, where applicable:
- Accession codes, unique identifiers, or web links for publicly available datasets
- A list of figures that have associated raw data
- A description of any restrictions on data availability

All data are available from the corresponding author on reasonable request.

Field-specific reporting

Please select the one below that is the best fit for your research. If you are not sure, read the appropriate sections before making your selection.

☒ Life sciences ☐ Behavioural & social sciences ☐ Ecological, evolutionary & environmental sciences

For a reference copy of the document with all sections, see nature.com/documents/nr-reporting-summary-flat.pdf

Life sciences study design

All studies must disclose on these points even when the disclosure is negative.

Sample size	Sample sizes were as large as possible given the experimental systems used.
Data exclusions	Data were not excluded.
Replication	All experiments were carried out a minimum of three times.
Randomization	Randomization was not necessary, the appropriate controls were used.
Blinding	Authors were blinded to the generation of simulation data, which were conducted by ANSYS-Fluent.

Reporting for specific materials, systems and methods

We require information from authors about some types of materials, experimental systems and methods used in many studies. Here, indicate whether each material, system or method listed is relevant to your study. If you are not sure if a list item applies to your research, read the appropriate section before selecting a response.

Materials & experimental systems

n/a	Involved in the study	
☐	☒	Antibodies
☒	☐	Eukaryotic cell lines
☒	☐	Palaeontology
☒	☐	Animals and other organisms
☒	☐	Human research participants
☒	☐	Clinical data

Methods

n/a	Involved in the study	
☒	☐	ChIP-seq
☒	☐	Flow cytometry
☒	☐	MRI-based neuroimaging

Antibodies

Antibodies used

Antibody:Anti-Rad53
Manufacturer: Abcam
Clonality: Rabbit Polycolonal
Cat number:ab104232

Antibody: Anti-Rad52
Gift from Dr. B Pfander, self-made
Clonality: Rabbit Polyclonal
Reference: Sacher et al. Nature Cell Biology (2006).

Antibody: Anti-Actin
Manufacturer: Thermo Fisher Scientific
Clonality: Mouse Monoclonal
Cat number: ma1744

Validation

Anti-Rad53: Antibody was validated via western blot using saccharomyces cerevisiae lysates. Untreated and phleomycin treated cells were tested for Rad53 signal and phosphorylation signal.

Anti-Rad52:The lab that generated this antibody confirmed that it detects Rad52. There are two cross-reactive proteins visible around 30kDa and 50 kDa at longer exposures.

Anti-Actin: This antibody has shown cross-reactivity with actin 1, 2, 3, 4, 7, 8, 11 and 12. MA1-744 has successfully been used in Western blot and ELISA procedures. By Western blot, this antibody detects a 45 kDa protein representing actin.
